# Structural Analysis Uncovers Lipid-Binding Properties of Notch Ligands

**DOI:** 10.1016/j.celrep.2013.10.029

**Published:** 2013-11-14

**Authors:** Chandramouli R. Chillakuri, Devon Sheppard, Ma. Xenia G. Ilagan, Laurie R. Holt, Felicity Abbott, Shaoyan Liang, Raphael Kopan, Penny A. Handford, Susan M. Lea

**Affiliations:** 1 Department of Biochemistry, University of Oxford, South Parks Road, Oxford OX1 3QU, UK; 2 Sir William Dunn School of Pathology, University of Oxford, South Parks Road, Oxford OX1 3RE, UK; 3 Department of Developmental Biology and Department of Medicine, Washington University School of Medicine, Euclid Avenue, St. Louis, MO 63110, USA

## Abstract

The Notch pathway is a core cell-cell signaling system in metazoan organisms with key roles in cell-fate determination, stem cell maintenance, immune system activation, and angiogenesis. Signals are initiated by extracellular interactions of the Notch receptor with Delta/Serrate/Lag-2 (DSL) ligands, whose structure is highly conserved throughout evolution. To date, no structure or activity has been associated with the extreme N termini of the ligands, even though numerous mutations in this region of Jagged-1 ligand lead to human disease. Here, we demonstrate that the N terminus of human Jagged-1 is a C2 phospholipid recognition domain that binds phospholipid bilayers in a calcium-dependent fashion. Furthermore, we show that this activity is shared by a member of the other class of Notch ligands, human Delta-like-1, and the evolutionary distant *Drosophila* Serrate. Targeted mutagenesis of Jagged-1 C2 domain residues implicated in calcium-dependent phospholipid binding leaves Notch interactions intact but can reduce Notch activation. These results reveal an important and previously unsuspected role for phospholipid recognition in control of this key signaling system.

## Highlights

• Notch ligands contain an N-terminal C2 phospholipid recognition domain
• Ca^2+^-dependent lipid binding by Jagged 1 is required for optimal Notch activation
• Lipid binding by Notch ligands is calcium dependent
• Notch ligands require bound calcium at the N terminus for full activity

## Graphical Abstract

**Figure:**
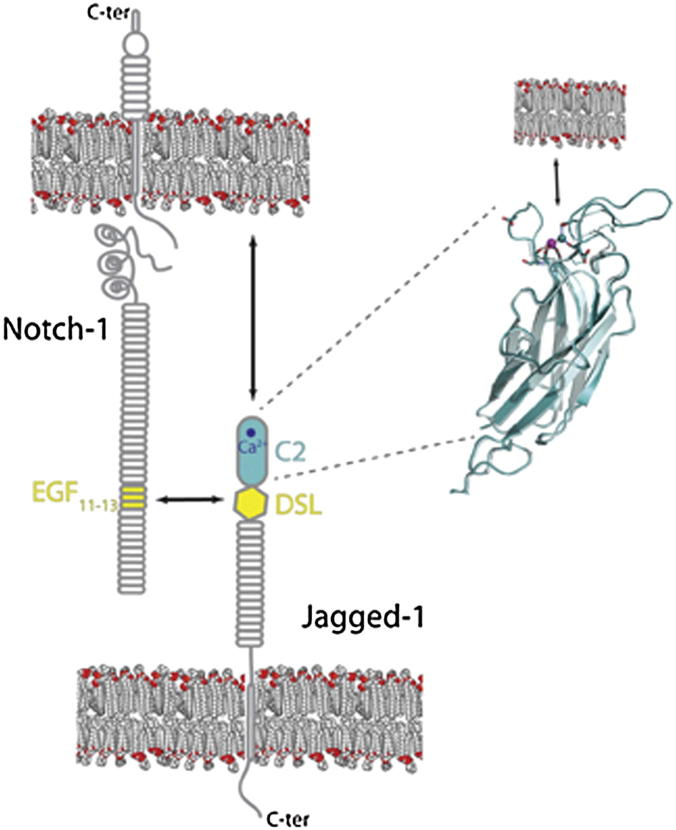

## Teaser

Handford, Lea, and colleagues now discover that the N-terminal portion of Notch ligands consists of a functional, calcium-dependent phospholipid recognition domain, the C2 domain. They also show that Notch-driven activation requires C2 domain activity in addition to canonical Notch-ligand binding.

## Introduction

The Notch signaling system has a crucial impact on the development and homeostasis of most tissues and organs (Harper et al., 2003). Dysregulation of the pathway results in a number of inherited and acquired disorders, including various cancers, and thus it is a key target for therapeutic intervention (Groth and Fortini, 2012). Despite the importance of this pathway, little is known about the structural basis of Notch receptor-ligand interactions and the molecular mechanisms that transduce this recognition event into signal activation (Bray, 2006; Chillakuri et al., 2012). The Notch receptor exists as a heterodimeric transmembrane protein that upon binding to ligands from Jagged/Serrate or Delta-like families results in the metalloprotease-cleaved Notch extracellular domain. A final intramembrane cleavage by gamma secretase releases the intracellular domain of Notch (NICD), which translocates to the nucleus and binds to a transcription factor of the CBF1, Suppressor of Hairless, Lag-1 (CSL) family. This complex, together with the coactivator MAML, relieves repression and activates genes of the *Hes* and *Hey* repressor families (Gordon et al., 2008). Interactions with the Notch receptor can activate or inhibit Notch signaling, depending on whether ligands are presented to Notch on adjacent cells (*trans*) or on the same cell (*cis*) (Sprinzak et al., 2010). O-glycosylation of Notch also plays an important role in regulating Notch signaling in a ligand-specific manner through the action of Fringe glycosyltransferases (Stanley and Okajima, 2010).

Direct contact between the epidermal growth factor (EGF)-like domain EGF12 of Notch and the Delta/Serrate/Lag-2 (DSL) domain, approximately 150 residues from the N terminus of the ligands, confers specificity to the interaction (Cordle et al., 2008a, 2008b; Whiteman et al., 2013). The role of the very N-terminal portion of the ligands is unknown, although its importance is implied by the fact that all Notch ligands through evolution position the DSL domain C-terminal to this region, and deletion of this region in a *Caenhorhabditis elegans* ligand, LAG-2, was shown to abolish its function (Henderson et al., 1997).

## Results

**Figure 1:**
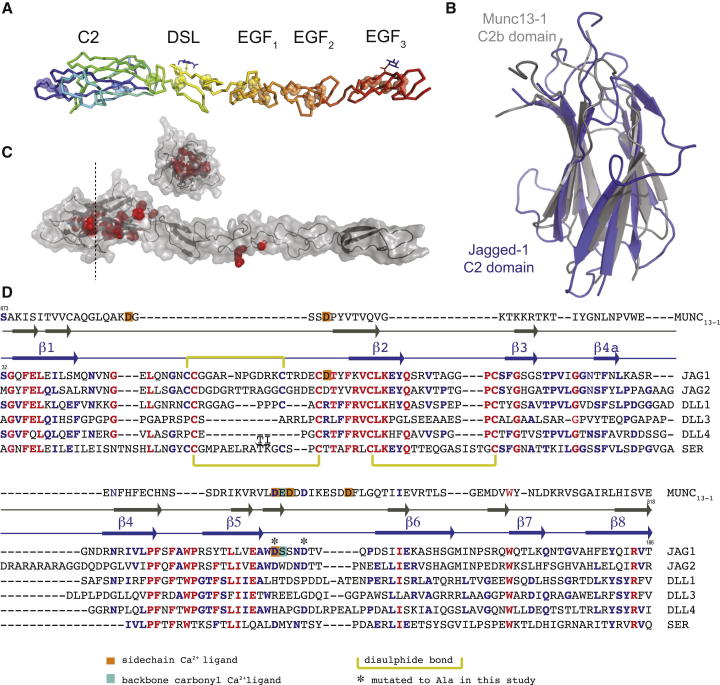
The Structure of Jagged1_N-EGF3_ Reveals a C2 Domain N Terminal to the DSL Motif (A) The structure of J-1_N-EGF3_ is shown in ribbon representation, with colors ranging from blue at the N terminus to red at the C terminus. Disulphide bonds are shown as sticks within semitransparent spheres, and the two glycans are shown as blue sticks. (B) Overlay of the N-terminal C2 domain of J-1_N-EGF3_ (blue) on the Munc13-1 C2b domain (gray). (C) Alagille-syndrome-associated missense mutations that affect the C2 domain (red) are mapped onto the structure of J-1_N-EGF3_ (gray cartoon and solid surface) and are located within the core of the domain (inset panel shows section at the level indicated by dotted line). (D) Structure-based sequence alignment of the Munc13-1 C2b domain and the N-terminal domains of Notch ligands. Most sequences associated with β strands contain some regions of absolute (red) or high (blue) sequence homology, whereas loop regions are more variable. Residues that coordinate the Ca^2+^ ion within the Munc13 domain are indicated, as are candidate Ca^2+^ ligand residues in J-1 mutated in this study (^∗^). See also Figures S1 and S2.

We expressed and solved the structure of a Notch ligand fragment consisting of the full N-terminal extension, the Notch-binding DSL domain, and the three adjacent EGF domains from human Jagged-1 (J-1_N-EGF3_) (Figure 1A; Table 1). Our structure demonstrates that the N-terminal portion of Jagged-1 folds as a C2-phospholipid-recognition domain with strong structural, but not sequence, homology to the Munc13 C2B domain (root-mean-square deviation [rmsd] 2.7 Å versus Protein Data Bank 3KWT, 10% sequence identity; Figure 1B; Cho and Stahelin, 2006; Shin et al., 2010). The C2 domain packs closely on top of the Notch-binding DSL domain without altering the structure of the DSL-EGF_1-3_ portion of the construct in comparison with our earlier structure for this region in isolation (Figure S1), and therefore extends the near-linear domain organization (Cordle et al., 2008a). Many missense mutations that affect this domain of J-1 cause Alagille syndrome, a developmental disorder that is usually associated with J-1 haploinsufficiency, and mapping these mutations onto our structure suggests that they destabilize the hydrophobic core and prevent correct folding of the C2 domain (Figure 1C; Penton et al., 2012). This is supported by our attempts to express J-1_N-EGF3_ constructs containing these Alagille-syndrome-associated substitutions, which largely resulted in no or little protein secretion (Figure S2), suggesting that these mutations lead to misfolding of the C2 domain and endoplasmic reticulum retention of J-1.

**Figure 2:**
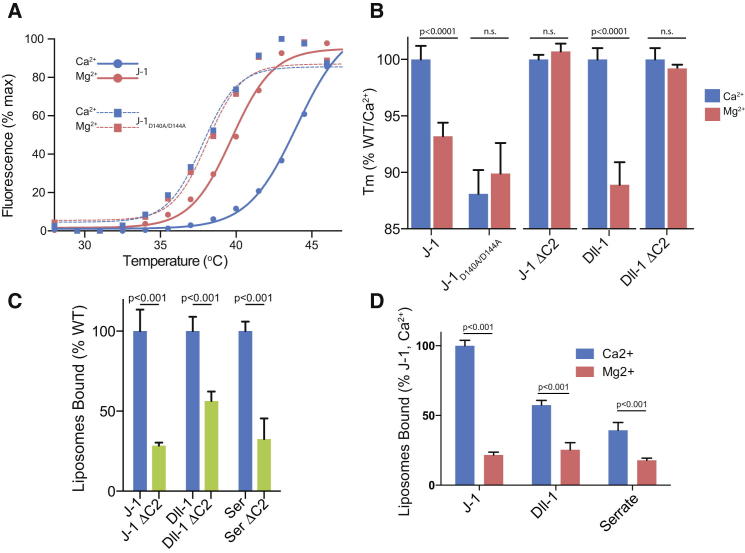
Notch Ligands Contain a Functional Ca^2+^-Dependent Phospholipid Domain at Their N Termini (A) Representative Thermofluor thermal denaturation curves reveal that WT J-1_N-EGF3-Fc_ shows a shift in T_m_ to a higher temperature in the presence of 2 mM CaCl_2_ (blue), but not 2 mM MgCl_2_ (pink). This shift is not seen in the D140A/D144A double mutant. Error bars are ± SD. (B) Notch-ligand proteins containing a WT C2 domain show a calcium-dependent shift in T_m_, whereas the D140A/D144A J-1 mutant or ΔC2 domain ligands do not. T_m_ values were extracted from thermal denaturation data using a Boltzmann sigmoidal fit. Means and SDs for five (J-1) or three (Dll-1) independent repeats are reported. (C) Plate-based assays (see Experimental Procedures) show that N-EGF3 constructs of three diverse Notch ligands (J-1, Dll-1, and *Drosophila* Serrate) all bind liposomes made from a PC/PS/PE mixture, whereas ΔC2 domain ligands do not. (D) Using the same assay, all three ligands are Ca^2+^ dependent in liposome binding, but they differ in their affinity for liposomes of a constant composition. See also Figures S3–S5.

Sequence alignments with other Notch ligands (Figure 1D) suggest that the C2 domain is present in all mammalian Notch ligands, with conserved residues mapping to those that play key structural roles, and residues exposed on the surface of the domain being more variable. Even evolutionarily distant Notch ligands such as *Drosophila* Serrate and the *C. elegans* LAG-2 retain N-terminal regions of similar lengths, and their sequences are compatible with a C2 fold. These data suggest an important role for the ligand C2 domain in the Notch signaling pathway, although the high level of variation in the loops connecting the key secondary structural elements also implies functional diversity. Since the strongest structural homology of the J-1 C2 domain is to calcium-dependent phospholipid-binding domains (e.g., the Munc13-1 C2b domain; Shin et al., 2010), we investigated whether J-1_N-EGF3_ binds Ca^2+^. We first used a limited proteolysis assay, which showed that Ca^2+^ (but not Mg^2+^ or EDTA) protects the J-1_N-EGF3_ fragment (but not a J-1 fragment lacking the C2 domain) from tryptic digestion. This suggests the presence of previously unrecognized Ca^2+^-binding sites (Figure S3), occupancy of which increases the degree of structure in the C2 domain. This is consistent with published data demonstrating that C2 domain loops involved in Ca^2+^ coordination adopt a more structured conformation in the presence of Ca^2+^ (Shin et al., 2010). To further probe Ca^2+^-dependent structural changes, we investigated thermal denaturation at constant ionic strength in the presence or absence of Ca^2+^ using an Fc-fused form of J-1. Comparisons of thermal denaturation profiles for protein fragments with (J-1_N-EGF3_)/without the C2 domain (J-1_ΔC2_) are complex, but suggest that this domain unfolds first in an event that is separable from unfolding of the other domains (Figure S4). Subsequent fitting of the first unfolding event using a Boltzmann sigmoidal model allows derivation of the temperature at the midpoint, or T_m_. This first melt T_m_ is significantly shifted to higher temperatures (Figures 2A and 2B) in the presence of calcium for J-1_N-EGF3_, but not for J-1_ΔC2_, providing strong evidence of a Ca^2+^-dependent increase in structure (shown by the increased heat required to induce unfolding). Since sequence comparisons implied conservation of the C2 domain in other Notch ligands (Figure 1D), we repeated these assays with human Delta-like 1 (Dll-1, 35% identity in the C2 domain) and found that Dll-1_N-EGF3_ also demonstrated a calcium-dependent shift in the first melt T_m_, whereas Dll-1_ΔC2_ did not. These data support the idea that the C2 domain is also present in Dll-1, as suggested by sequence alignments, and binds Ca^2+^ despite the high level of sequence variation in the putative calcium-binding loops, which makes it difficult to predict which residues are likely to coordinate the metal ion.

We then sought to establish whether the Jagged C2 domain is functionally active as a phospholipid-binding domain. We used both solution- and plate-based assays to study protein binding to fluorescently labeled liposomes consisting of mixtures of phosphatidylcholine/phosphatidylserine/phosphatidylethanolamine (PC/PS/PE). These assays demonstrated that the J-1_N-EGF3_, but not the J-1_ΔC2_, construct bound liposomes in a Ca^2+^-dependent manner (Figures 2C and S5), supporting our structure-based hypothesis that the C2 domain at the N terminus of Jagged-1 is a functional phospholipid-recognition domain under physiological conditions. Human Dll-1 and *Drosophila* Serrate (40% identity in the C2 domain) were also investigated and were found to bind liposomes in a Ca^2+^- and C2-domain-dependent fashion (Figure 2C). However, as expected from sequence diversity in the ligand-binding loops, a comparative analysis of these three ligand constructs showed different levels of liposome binding (Figure 2D).

To further dissect the functional consequences of phospholipid recognition, we designed a series of site-directed J-1_N-EGF3_ mutants targeting aspartate residues within the loops homologous to those involved in Ca^2+^ and phospholipid binding in Munc13-1 C2b (Shin et al., 2010; Figure 1D). Structural and sequence comparisons suggested that aspartate residues in the β5-β6 loop (D140 and D144) were most likely involved in Ca^2+^ coordination (located in structurally equivalent positions to residues D757 and D759 in the Munc13-1 C2b domain and conserved through human Notch-ligand proteins). However, we could not rule out the possible involvement of aspartates in the β1-β2 loop, which are less conserved through human Notch-ligand proteins. We therefore cloned and expressed single and double mutants of J-1_N-EGF3_ with alanine substitutions in place of aspartates in the β5-β6 loop. Many of these mutations led to reduced protein stability and could not be further studied; however, the D140A/D144A double mutation within the β5-β6 loop was compatible with production of stable protein. Analysis by tryptic digestion (Figure S3) and thermal denaturation (Figures 2A and 2B) assays demonstrated that this mutant has lost the ability to bind calcium at the concentrations used in our functional assays.

**Figure 3:**
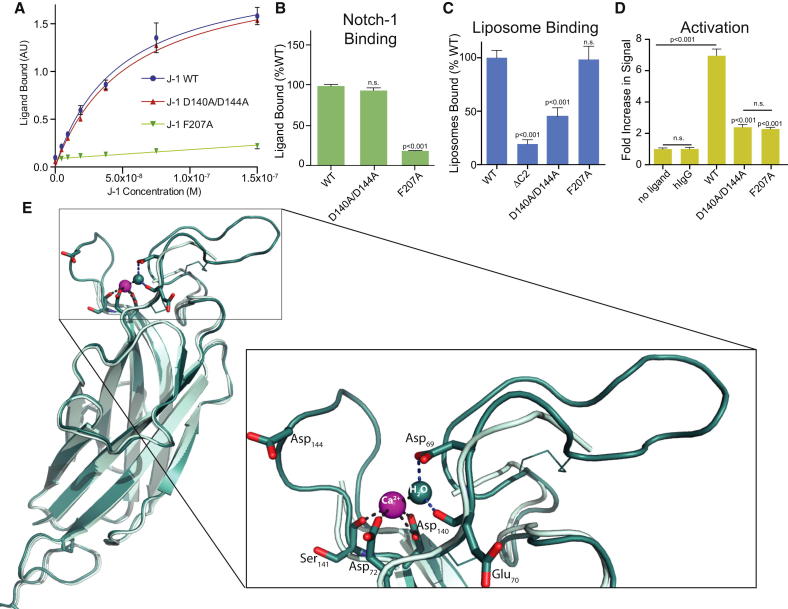
Mutation of Residues within the β5-β6 Loop of the Jagged-1 C2 Domain Leaves Notch Binding Intact but Disturbs Phospholipid Binding and Notch Activation (A and B) Notch-1 binding of WT J-1_N-EGF3_ and mutants assessed by ELISA. Substitution F207A (within the Notch binding face of the DSL domain; Cordle et al., 2008a) reduces binding to background levels, whereas D140A/D144A double substitution in the β5-β6 loop leaves binding intact. (C) Liposome binding by WT J-1_N-EGF3_ and mutants assessed using fluorescently labeled liposomes (PC/PE/PS). (D) The ability of WT J-1_N-EGF3_ F207A control and the D140A/D144A mutant to activate receptor in Notch1-transfected cells was assessed using a split luciferase reporter system. (E) Structure of WT J-1_N-EGF3_ at pH 7.5 in the presence of 7.5 mM Ca^2+^ reveals a single calcium coordinated between the β5-β6 and β1-β2 loops. J-1 is shown as a cartoon representation (cyan, apo structure; teal, Ca^2+^-bound form copy A) with key residues highlighted as stick representations and the bound Ca^2+^ (pink) and a water molecule (teal) involved in coordination shown as spheres. Note the bidentate coordination by D140, which explains the loss of Ca^2+^ dependence associated with the D140A/D144A double mutant. All error bars are ± SD. See also Figures S1 and S5.

We next investigated whether the J-1 D140A/D144A substitutions affected Notch activation. An ELISA-based Notch-binding assay first demonstrated that this double mutant was competent for Notch-1 binding, in contrast to a previously identified ablative DSL domain mutant (F207A) control (Figures 3A and 3B). By contrast, F207A was wild-type (WT)-like in its ability to bind liposomes, while D140A/D144A showed significantly reduced liposome binding (Figures 3C and S5) close to the background level of binding seen in a ΔC2 construct. We next used a quantitative split luciferase Notch activation reporter system (Figure 3D) in which Notch-1 was expressed on the cell surface and Fc fusions of Jagged-1 were presented on the well surface (to mimic Notch-ligand interactions in *trans*). This assay was chosen because it utilizes a receptor-proximal reporter whose output is directly proportional to the amount of NICD that is released (Ilagan et al., 2011), and thus is well suited for quantifying the extent of a deficit caused by ligand mutations. Wild-type J-1_N-EGF3_ was able to promote Notch activation in this assay, whereas the F207A mutant, which cannot bind Notch, was strongly impaired. Importantly, the double mutant D140A/D144A ablated Notch-dependent activation to a level similar to that observed for F207A, even though this mutant is competent for Notch-1 binding in vitro.

To explore the molecular basis of the effects of this double mutant, we determined the J-1_N-EGF3_ structure from crystals grown at a physiological pH in the presence of 7.5 mM Ca^2+^ (Table 1). These crystals contain two independent copies of J-1_N-EGF3_, and, as observed for other Ca^2+^-dependent C2 domains, Ca^2+^ is seen to bind in a cleft between the β1-β2 and β5-β6 loops at the top of the C2 domain (Figures 3E and S1), ordering them despite the increased length of these loops in Jagged-1 compared with other C2 domains. Both copies of the molecule bind a single Ca^2+^ ion, coordinated by the side chains of Asp72 (OD1) and Asp140 (OD1 and OD2) and the main-chain carbonyl of Ser141 (Figure 3E, inset). The observed bidentate coordination of the bound Ca^2+^ by Asp140 suggests that it is substitution of this side chain by Ala in the D140A/D144A mutant that explains the loss of Ca^2+^ binding as shown by increased proteolytic susceptibility, reduced T_m_, and significantly reduced phospholipid binding. It is noteworthy that even in the presence of Ca^2+^ at concentrations that our thermal denaturation assay suggests would be fully saturating (Figure S4), the β5-β6 and β1-β2 loops remain highly mobile, with their conformations varying between the two crystallographically independent copies of the molecule (Figure S1). This variation in loop structure leads to differences in how the Ca^2+^ coordination shell is completed in these independent copies, and therefore we cannot exclude the possibility that a further rearrangement of the β5-β6 loop leads Asp144 to be directly or indirectly (via Ca^2+^ coordination) involved in phospholipid binding.

## Discussion

Collectively, our molecular and cellular data strongly imply that in addition to the Notch-1/Jagged-1 *trans* interaction, signaling may also require Ca^2+^-dependent phospholipid binding by Jagged-1. Our experimental data demonstrating that this activity is found in other Notch ligands (Figure 2), together with the observed sequence conservation (Figure 1), further suggest that all Notch signaling will have a lipid-binding element. It is of interest that three disease-causing mutations associated with extrahepatic biliary atresia map to the loop structures of the C2 domain (Figure S1) rather than to the hydrophobic core typical of Alagille syndrome mutations, emphasizing the importance of this region for function.

Notch-dependent signaling is a complex phenomenon involving a series of extra- and intracellular events that are required for signal generation under tightly controlled circumstances. Additional complexity was recently added to the Notch signaling pathway when a Notch ligand, Jagged-1, was demonstrated to interact with and be sequestered from productive Notch interactions by another cell-surface protein, CD46 (Le Friec et al., 2012). Our structural and functional data now add another component to this complex pathway, suggesting that C2-domain-mediated lipid binding is a modulator of the signaling process. Much further work will be required to dissect this biology. It is unlikely that the generic phospholipids we used to assay binding are the functionally critical ones for Jagged-1, and indeed an earlier genetic study implicated glycosphingolipids in modulating ligand activity in *Drosophila* (Hamel et al., 2010). Furthermore, the lack of conservation among the loop residues within the ligand C2 domains implies that although they are all likely to be lipid-binding domains, they are likely to vary in their affinity or specificity for different lipids. The structural similarities to C2 domains involved in synaptic exocytosis, where, in addition to the Ca^2+^-dependent phospholipid-binding site, there is also a second site located on the surface of strands β3 and β4 (the cationic β-groove) that putatively allows these domains to play roles in membrane penetration and/or orientation of proteins relative to the membrane are intriguing (Cho and Stahelin, 2006). Whether or not a second binding site exists within the Notch-ligand C2 domains remains an open question (Cho and Stahelin, 2006). Identification of this unexpected but important component of Notch signaling therefore opens up many new avenues for future work on the involvement of lipid biochemistry, including the identification of functionally relevant lipids in ligand/receptor-presenting cells, and characterization of potential roles of the C2 domain in Notch-ligand biology.

## Experimental Procedures

### Protein Production

Jagged and Notch proteins were produced in human embryonic kidney 293T (HEK293T) cells using a transient transfection system (Aricescu et al., 2006). Proteins secreted into the medium were purified using Ni-chelating sepharose followed by gel filtration. Protein used for crystallization was produced in 293S GnTI^−/−^ cells, which have been shown to lack complex N-glycosylation and thus improve the homogeneity of the protein.

### Notch Binding Assay

For the Notch binding assay, 200 ng of human Notch-1_N-EGF14_ was coated overnight at 4°C per well of a MaxiSorp plate. Wells were blocked using 2% gelatin, 1% milk in Tris-buffered saline with Tween 20 (TBS-T). WT and mutant Fc-tagged constructs of J-1_N-EGF3_ were added at the required concentrations (100 nM in general) and antihuman immunoglobulin G Fc antibody-horseradish peroxidase conjugate (Sigma) was used to detect binding.

### Thermofluor Thermal Denaturation Assay

Thermofluor thermal denaturation assays were performed to measure Ca^2+^-induced structural changes (Jakobi et al., 2011) in 50 mM Tris-HCl (pH 7.5), 200 mM NaCl with 2 mM Ca^2+^/Mg^2+^ (J-1 assays), or 10 mM Ca^2+^/Mg^2+^ (DLL-1 assays) with ∼200 μg Notch ligand proteins as Fc fusions per well, using Sypro Orange (Sigma-Aldrich) at a dilution of ∼1:300. Assays were performed on a Stratagene Mx3005P using steps of 1.5°C over 90 s from 25°C to 77.5°C for the number of repeats specified. All data were normalized and fit using a Boltzmann sigmoidal function in GraphPad Prism 6.0 to extract T_m_ values.

### Limited Proteolysis Assay

For limited proteolysis assays, 10 μg of WT and mutant histidine-tagged J-1_N-EGF3_ proteins were treated with trypsin (protein:trypsin 50:1) in the presence of 10 mM Ca^2+^/Mg^2+^ or EDTA in 50 mM Tris pH 7.5, 200 mM NaCl buffer. Reactions were incubated at 37°C and samples were collected at different time intervals and analyzed by SDS-PAGE.

### Phospholipid Binding Assay

Liposomes were prepared using PC/PS/PE-fluorescein (80:15:5) by ultrasonication/extrusion. The bead-based assay was performed using 10 μl of Protein A-sepharose beads immobilized with 10 μg of Fc-fused protein per reaction. Beads were incubated for 30 min with 1 μM phospholipids in 20 mM HEPES pH 7.5, 200 mM NaCl containing 2 mM Ca^2+^/Mg^2+^/EDTA. In the high-throughput plate-based assay, equimolar concentrations of proteins (400 nM) were coated on a MaxiSorp plate. After blocking with 1% gelatin, 100 μM of phospholipids was added in the presence of 2 mM free Ca^2+^/Mg^2+^ or 0.5 mM EGTA. In both methods, after washing with relevant buffers, bound liposomes were solubilized with 0.3% Triton X-100 and fluorescence was measured using a NanoDrop 3300.

### Crystallization and Structure Solution

Crystals for the 2.5 Å apo structure were grown in 0.3 μl sitting drops using vapor diffusion at 3.2 mg/ml with 0.97 M sodium citrate (pH 5.0), 19.4% polyethylene glycol 6000 mother liquor at 25% after initial screening using commercially available reagents from Molecular Dimensions. Diffraction data were collected at the ESRF on beamline ID29 with a Pilatus 6M-F detector. The structure was solved by molecular replacement using the program Phaser and the previously published structure of Jagged1_DSL-EGF123_ (Cordle et al., 2008a; McCoy et al., 2007). The C2 domain was modeled using a combination of autobuild, buccaneer, and manual modeling (Adams et al., 2010; Cowtan, 2006).

Both calcium-bound forms were crystallized at 5.0 mg/ml with commercial reagents from Molecular Dimensions using the sitting-drop vapor-diffusion method with a drop size of 0.2 μl and 25% mother liquor. The 2.38 Å form was crystallized with 0.2 M sodium acetate, 0.1 M Bis-Tris propane (pH 7.5), and 20% polyethylene glycol 3350. The 2.84 Å form was crystallized using 0.2 M sodium iodide, 0.1 M Bis-Tris propane (pH 6.5), and 20% polyethylene glycol 3350. In addition, 10 mM CaCl_2_ was added to the initial protein solution, and the final concentration in the drop was 7.5 mM. Data were collected at the Diamond Synchrotron facility on beamline I03 with a Pilates 6M-F detector. Phases were determined by molecular replacement using the apo structure and the program Phaser. Refinement for all structures was performed using autobuster and COOT (Emsley et al., 2010; Smart et al., 2012). The density for the newly ordered loops is shown in Figure S1 prior to rebuilding. Data refinement and model statistics are summarized in Table 1.

### Notch Activation Assays

Purified J-1_N-EGF3_ Fc-fusion proteins (10 μg/ml in TBS) were immobilized on culture plates overnight at 4°C. The plates were washed once with TBS and then the Notch split luciferase reporter cells (HelaTetON cells stably expressing Notch1-NLuc and CLuc-RBPjκ) were seeded onto the immobilized ligand in the presence of 0.5 μg/ml doxycycline to induce reporter expression. After 24 hr, the cells were assayed for bioluminescence as previously described (Ilagan et al., 2011).

## Figures and Tables

**Table 1:** Crystallographic Data Collection and Refinement Statistics

	Apo	Ca^2+^	Ca^2+^
Wavelength (Å)	0.97625	0.976	0.976
Space group	P 1	P 1	P 1
Cell dimensions			
a, b, c (Å)	56.93, 59.84, 62.94	55.98, 60.25, 62.65	55.95, 60.56, 63.03
α, β, γ (°)	95.04, 101.90, 98.70	92.59, 104.44, 106.67	92.89, 104.95, 105.92
Resolution (Å)	61.12–2.50 (2.62–2.50)	60.19–2.38 (2.40–2.38)	60.38–2.84 (3.01–2.84)
Redundancy	1.84 (1.79)	1.77 (1.78)	2.45 (2.41)
Completeness (%)	82.9 (38.3)^a^	97.3 (96.2)	98.2 (97.8)
I/sigma (I)	3.7 (0.7)	4.3 (1.6)	11.3 (1.8)
Rmerge (%)	11.8 (54.2)^a^	14.4 (46.0)	15.9 (45.5)
Refinement statistics			
Resolution limit (Å)	2.50	2.38	2.84
Reflections	22,816	31,595	17,666
R_work_/R_free_ (%)	20.4/24.6	21.8/24.6	19.6/24.9
Residues in allowed regions of Ramachandran plot (%)	99.6	99.6	100.0
Residues in most favored regions of Ramachandran plot (%, Å)	95.1	93.7	92.1
Rmsd bond lengths (λ)	0.008	0.01	0.01
Rmsd bond angles	1.12	1.19	1.23
Mean B value (Å^2^)	70.26	43.98	68.53
Protein	70.03	43.88	68.34
Glycosylation	95.54	69.15	94.36
Waters	49.36	35.45	39.49
Ions		58.68	54.47

Values denoted in brackets refer to highest-resolution shell.

a Refinement of the structure was performed at 2.5 Å, at 2.8 Å. Completeness (%) = 89%, and Rmerge (outer shell) = 30.0%.

## Accession Numbers

Coordinates and X-ray data sets have been deposited in the Protein Data Bank under accession numbers 4CC0, 4CC1, and 4CBZ.

## Supplemental Information

Document S1. Figures S1–S5

Document S2. Article plus Supplemental Information
