# Supplementary material for: Structural Analysis Uncovers Lipid-Binding Properties of Notch Ligands
Source: Cell Rep. 2013 Nov 14;5(4):861–7. doi: 10.1016/j.celrep.2013.10.029 (PMC3888931; doi:10.1016/j.celrep.2013.10.029)
Supplement: Document S1. Figures S1–S5 [file mmc1.pdf]

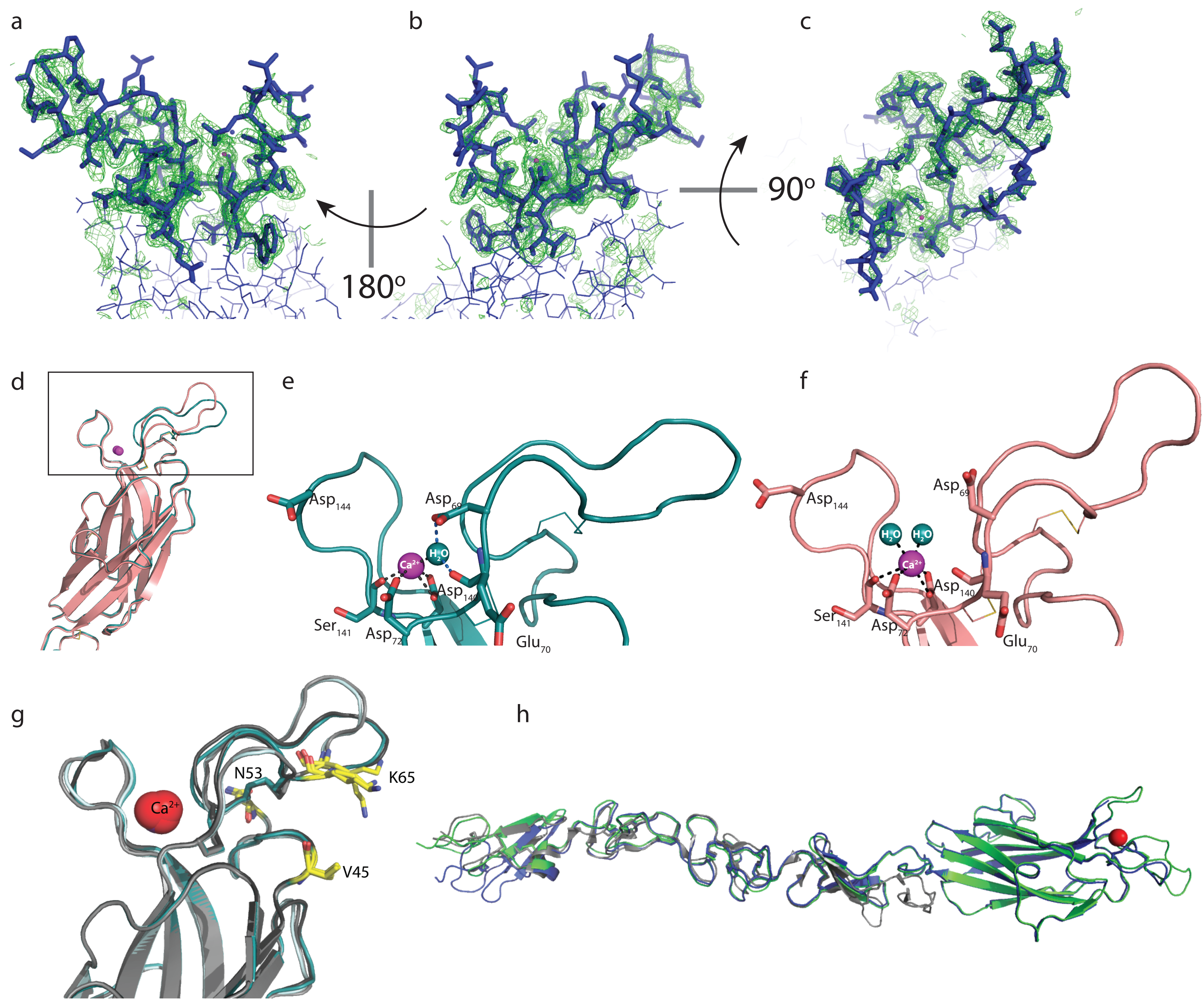

Supplemental Figure 1 related to Figures 1 and 3. Structural validation, detailed views and comparisons.
(**a-c**) $|F_O|-|F_C|$, αC map calculated using the model prior to first building residues B59-73, B139-150, the bound Ca^{2+} and associated waters shows clear density for the loops at the tip of the C2 domain that were disordered in the apo structure.
(**d-f**) Overlay of the calcium binding sites in the two independent copies of J-$1_{N\text{-}EGF3}$(d) overview of the C2 domain overlay - boxed region corresponds to zoom regions shown in other panels (e) zoom of copy A (as shown in Figure 3) (f) zoom of copy B. Coordination by the side chains of Asp_{72}, Asp_{140} and main chain of Ser_{141} are conserved between both molecules. Other details differ primarily due to rearrangement of the β1-β2 loop.
(**g**) Overlay of the DSL-distal surface of the Jagged-1 C2 domains from the four unique chains built in two different crystal forms (light/dark grey cartoon 2.8A form; light/dark cyan cartoon 2.4A) all with Ca^{2+} bound. The residues highlighted as yellow side chains are those in which mutations associated with extrahepatic biliary atresia are found.
(**h**) Overlay of cartoon of representative chains from all structures of Jagged-1 grey J-$1_{DSL\text{-}EGF3}$ (Cordle et al, NSMB, 2008); blue J-$1_{N\text{-}EGF3}$in the absence of Ca^{2+}; green J-$1_{N\text{-}EGF3}$ in the presence of Ca^{2+}

a

J1 mutation	Expression	Effect on protein
I152T	Yes	Misfolding -ER retained
Y181N	Yes	Misfolding -ER retained
R184H	Yes	Misfolding -ER retained

b

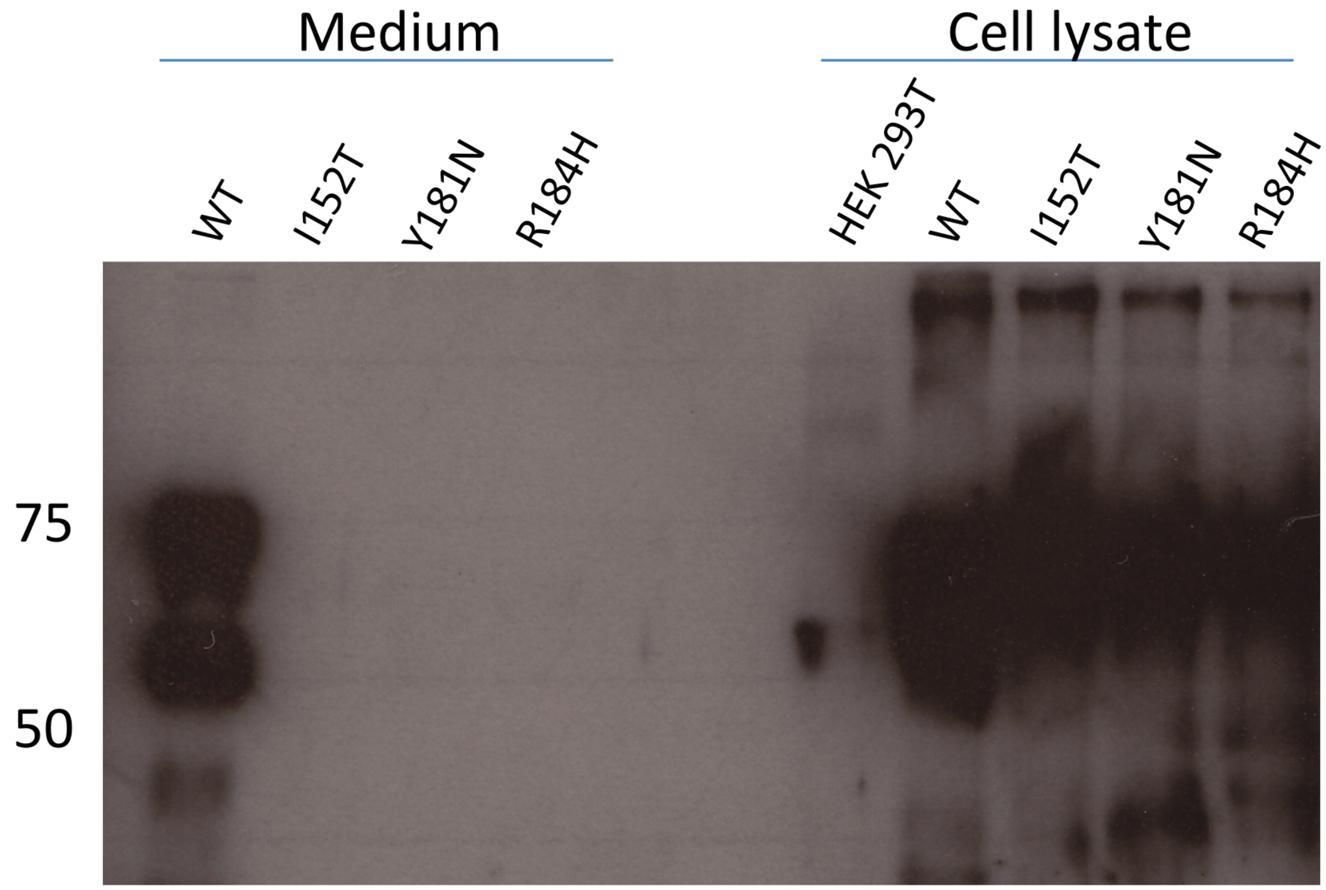

Supplemental Figure 2 related to Figure 1. Mutations associated with Alagille disease cause misfolding of Jagged-1 and its retention within the endoplasmic reticulum. (a) Alagille-mutations expressed and summary of the protein production phenotype (b) SDS-PAGE of extracellular medium and cell-lysate reveals that, with the exception of wild-type Jagged-1 which is secreted into the extracellular medium, the Alagille-associated mutations all result in retention of the protein expressed within the cell.

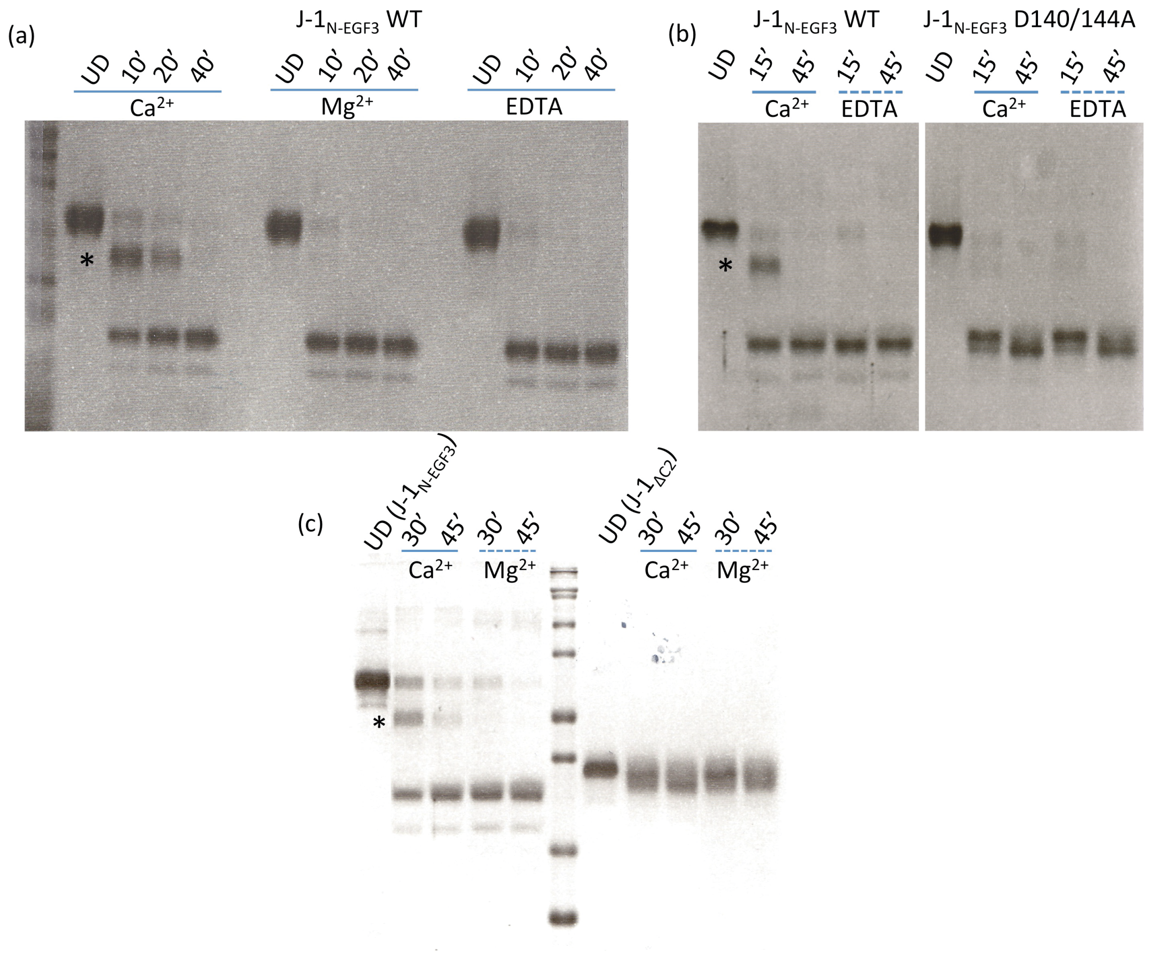

Supplemental Figure 3 related to Figure 2. Limited proteolysis of J-1 proteins. (a) $J\text{-}1_{N\text{-}EGF3}$ WT protein is protected against trypsin only in the presence of Ca^{2+} and not Mg^{2+} or EDTA. (b) Ca^{2+} dependent protection is lost in the D140A/D144A mutant indicating loss of Ca^{2+} binding. (c) only fragments with a C2 domain show this Ca^{2+} dependent protection. UD=undigested, * indicates the protected band.

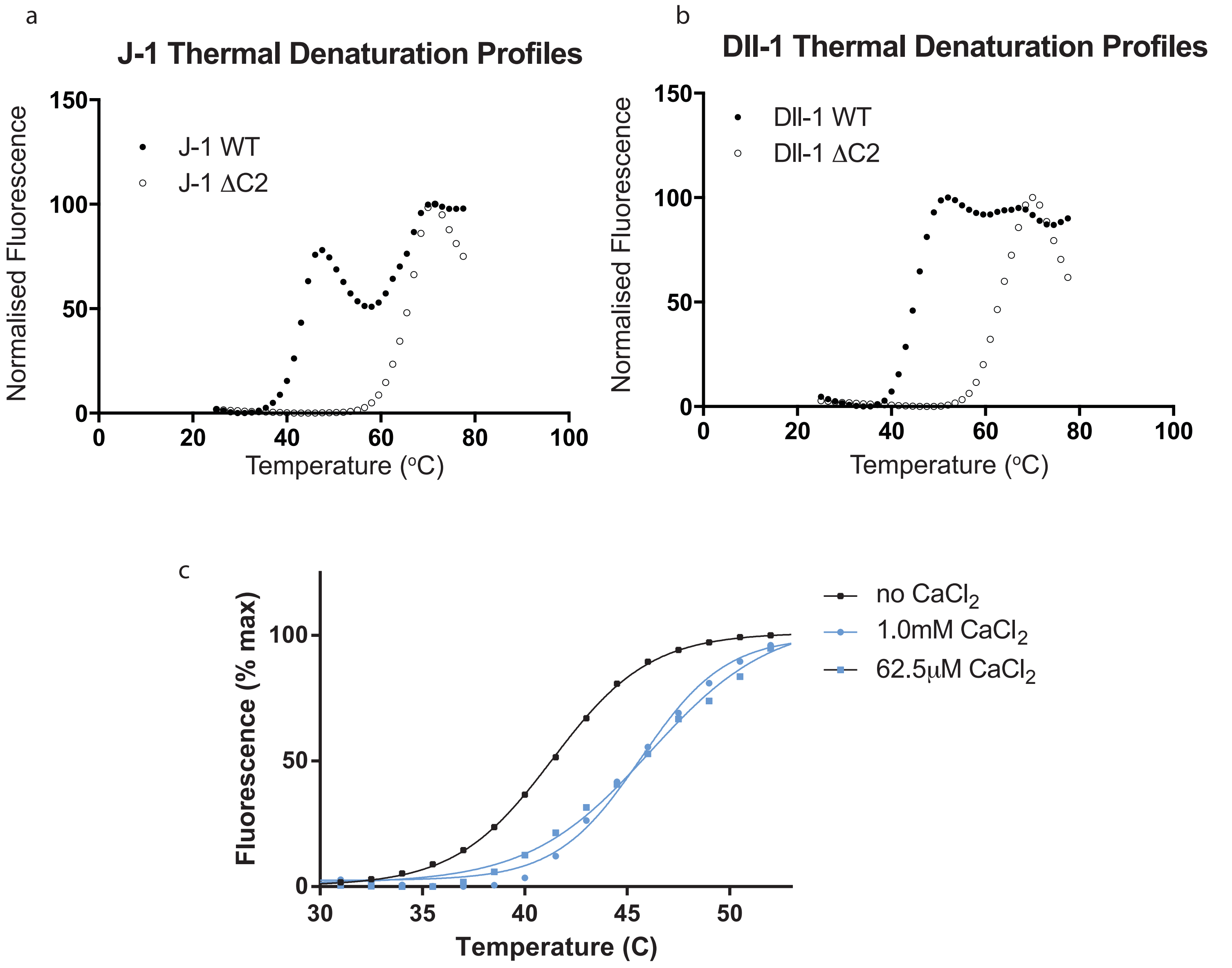

Supplemental Figure 4 related to Figure 2. Thermal denaturation profiles for Notch ligand protein constructs with and without the C2 domain (see methods) demonstrate that the C2 domain melts first and that the temperature of the first melt depends on the presences of Calcium (a) Profile for Jagged-1 with/without the C2 domain (b) profile for Delta-like 1 with/without the C2 domain (c) The temperature with which the C2 domain melts in Jagged-1 depends on the presence of Calcium with the shit to higher temperature occuring even at CaCl2 concentrations as low as 62.5μM implying the affinity of the C2 domain for calcium is below this level.

a

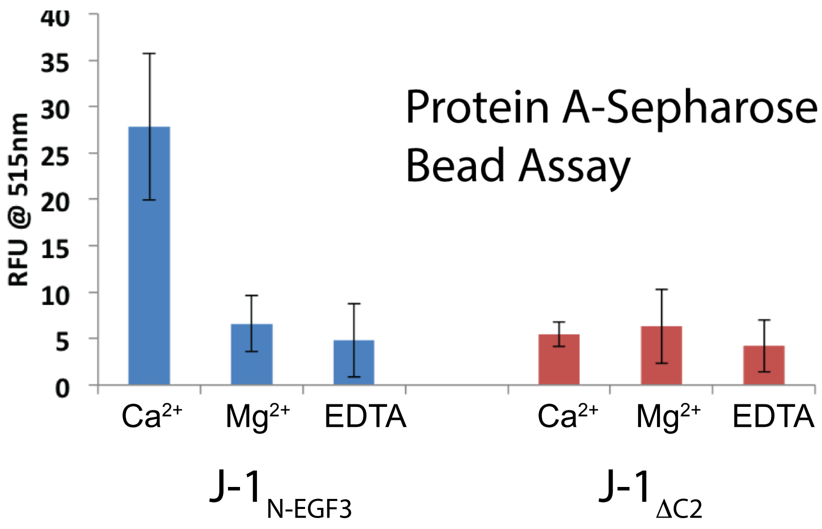

b

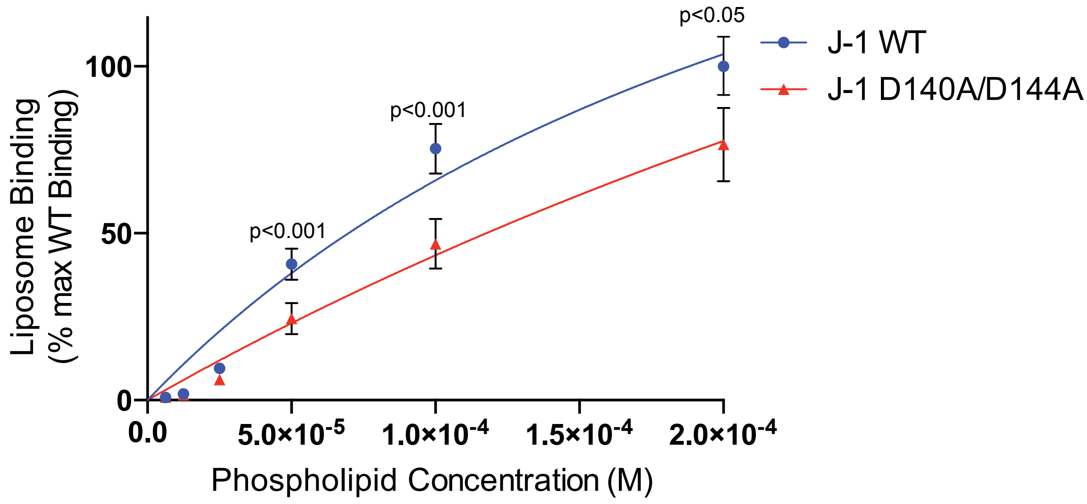

Supplemental Figure 5 related to Figures 2 and 3. Phospholipid binding by Jagged-1. (a) J-1 fragments containing the C2 domain, but not those lacking the C2 domain show Ca^{2+} dependent binding of liposomes using a bead-based assay (see methods). Data are shown as mean +/- standard deviation for n=5 independent repeats. (b) Titration of Phospholipid Binding at constant Jagged-1 concentration using the plate based assay (see methods) demonstrates that the D140A/D144A is significantly reduced in its ability to bind liposomes at a range of phospholipid concentrations.
